# Lactate modulates microglial M2 polarization *via* H3K9 lactylation in ischemic stroke

**DOI:** 10.1515/jtim-2026-0010

**Published:** 2026-03-26

**Authors:** Bingwei Li, Kan Xu, Jinlu Yu

**Affiliations:** Department of Neurosurgery, First Hospital of Jilin University, Changchun, Jilin Province, China

**Keywords:** ischemic stroke, histone lactylation, microglial polarization, Nrf2, neuroprotection

## Abstract

**Background and Objectives:**

Ischemic stroke triggers pathological neuroinflammation primarily mediated by microglial activation. However, the epigenetic impact of lactate, a metabolite that accumulates during cerebral ischemia, on this process has not been comprehensively investigated. This study aimed to determine whether lactate influences microglial polarization through histone lactylation during prolonged cerebral ischemia.

**Methods:**

*In vitro* models using lactate-treated BV-2 microglia and *in vivo* models of transient middle cerebral artery occlusion (MCAO) mice were established. Analyses were conducted at 4 to 12 h post-occlusion. Our comprehensive analysis included H3K9la-targeted CUT& Tag sequencing, Nrf2 promoter-specific ChIP-qPCR, flow cytometry for polarization markers, cytokine enzyme-linked immunosorbent assays (ELISAs), and neuronal viability assays.

**Results:**

Under ischemic conditions, lactate markedly increased H3K9 lactylation, with selective enrichment at Nrf2 promoters. This epigenetic modification resulted in a phenotypic shift toward anti-inflammatory M2 states in microglia, both *in vitro* and *in vivo*. Mechanistically, H3K9la activated the Nrf2/HO-1 pathway, effectively suppressing nuclear factor kappa-light-chain-enhancer of activated B cells (NF-κB) signaling and significantly reducing pro-inflammatory cytokine secretion. Importantly, conditioned medium derived from lactate-treated microglia mitigated the neurotoxic efects induced by microglia to some extent.

**Conclusion:**

Our findings suggest that lactate confers neuroprotection *via* epigenetic activation of Nrf2 *via* H3K9la, thereby polarizing microglia towards inflammation-resolving states. This finding uncovers a novel metabolic-epigenetic target for therapeutic intervention in ischemic stroke.

## Introduction

Stroke has emerged as a major public health concern with a high residual mortality rate that can be divided into ischemic stroke, which accounts for 84.4% of all cases, and hemorrhagic stroke.^[1, 2, 3]^ During ischemic stroke, temporary or permanent interruption of blood flow leads to dysfunction and metabolic disorders in local brain tissue. In addition, it can lead to programmed cell death, disrupt the balance of oxidative and antioxidant effects in the body, trigger excitotoxic effects from excitatory amino acids, and induce cellular inflammatory reactions.^[4, 5, 6]^

Microglia, the resident immune cells of the central nervous system, are activated prior to the infiltration of macrophages and have a significant impact on the pathological changes associated with cerebral ischemia. An increasing body of evidence suggests that microglia can monitor changes in the microenvironment of the central nervous system and play an essential role in immune activity and phagocytosis.^[7, 8, 9]^ The activated microglia can be divided into two main types, which are the “classically activated” M1 phenotype and the “alternatively activated” M2 phenotype.^[10,11]^ Studies have shown that M1 microglia are primarily characterized by CD86, IL-1β, and iNOS, which are associated with cytotoxic effects and the secretion of inflammatory cytokines.^[12,13]^ M2 microglia is now understood to exert anti-inflammatory effects and promote wound healing and tissue repair, with CD206 and Arg1 serving as key biomarkers.^[14,15]^ Activated microglia can also undergo functional transition between M1 and M2 types under specific environmental conditions, a process known as polarization.^[16]^

The distinct polarization phenotypes of microglia are pivotal in determining the progression of injury and subsequent neural recovery following ischemic stroke.^[17]^ For instance, the M2 microglial population significantly increases within 24 h post-acute cerebral ischemic injury, with their phenotype markers being highly expressed in the ischemic core.^[18]^ During this early phase, microglia are instrumental in mitigating neuronal damage and facilitating tissue repair.^[19]^ Subsequently, the M2 phenotype gradually transitions to the M1 phenotype, with microglial distribution expanding from the ischemic core to the penumbra. The expression of M1 phenotype markers begins to rise after 3 days of ischemia, peaking at 14 days.^[20]^ The prevalence of M1-type cells is most pronounced on day 7 and can persist for several weeks.^[20]^ During this period, microglia enhance the release of inflammatory factors, thereby exacerbating brain damage, which is detrimental to regeneration and repair in the later stages of cerebral ischemia.^[21]^ This progression illustrates that M2 microglia predominates in the early stages of cerebral ischemia, followed by a predominance of M1 microglia.

Lactate serves as a crucial signaling molecule in the development of ischemic stroke, potentially exerting neuroprotective effects by inducing microglia to transition into an M2 phenotype. Some studies have shown that the lactate levels in the infarcted area and ischemic penumbra are significantly elevated during the early stages of cerebral ischemic injury.^[22]^ Therefore, the significant accumulation of lactate is a key marker of cerebral ischemia/ hypoxia injury.^[23,24]^ Previous studies have reported that lactate concentrations in the ischemic brain can reach 15–40 mmol/ L within h of ischemia-reperfusion.^[25]^ Similarly, lactate concentrations of 18.2 ± 2.9 mmol/L in the hippocampal ischemic region of middle cerebral artery occlusion (MCAO) rats at 6 h postischemia was observed.^[23]^ In recent years, an increasing number of experimental results have demonstrated that lactate plays a crucial biological role in cells. The increase in extracellular lactate levels contributes to the acidification of the tumor microenvironment, increased angiogenesis, and immune signal suppression.^[26, 27, 28]^ In addition, lactate can induce tumor-associated macrophages to polarize towards the M2 phenotype and increase the expression of vascular endothelial growthfactor (VEGF).^[29]^ Moreover, lactate can alleviate neurological damage and reduce infarct volume in neonatal rats with cerebral ischemia.^[25]^ However, no reports have hitherto assessed the role and mechanism of high-concentration lactate in the activation of microglia at the site of cerebral ischemic injury.

Mounting evidence has established lactate as a pivotal metabolic-epigenetic regulator in cerebral ischemia, with histone lactylation emerging as a key link between dysregulated energy metabolism and pathological neuroinflammation. Under ischemic conditions, anaerobic glycolysis drives massive lactate accumulation in the ischemic core and penumbra—reaching concentrations up to 35 mmol/L in rodent models.^[25]^ It not only serves as an alternative energy substrate but also modulates gene expression *via* site-specific lactylation of histone lysine residues. Global profiling of lactylated proteins in cerebral endothelium from ischemia-reperfusion injury rats identified widespread lactylation of histones (H3K4, H3K9, H3K27) and non-histone proteins involved in vascular integrity and inflammatory signaling, highlighting the pervasiveness of this modification in ischemic brain pathology.^[30]^ In neurons, dysregulated H3K14 lactylation exacerbates ferroptosis by disrupting calcium homeostasis, underscoring cell-type-specific roles of histone lactylation (Kla) in ischemia.^[31]^

Mechanistically, the acetyltransferase p300 has been implicated as a critical mediator of lactate-driven Kla in ischemic brains. p300 is recruited to target gene promoters *via* lactylation-sensitive transcription factors and directly catalyzes histone lactylation, as demonstrated in astrocytes where p300-dependent lactylation of ARF1 mitigates mitochondrial dysfunction post-ischemia.^[32]^ p300 can interact with myogenic regulatory factors (MyoD/MyoG) and participate in histone acylation regulation near myogenic genes in the differentiation of myoblasts.^[33,34]^ The promoter motif recognized by H3K9la is consistent with the promoter binding motif of MyoD.^[35]^ This motif can recruit p300 to the promoter region of specific genes, such as Neu2 in myoblasts.^[36,37]^ The recruited p300 can promote lactylation modification of histones in the promoter region of target genes, thereby participating in cellular functional regulation. For example, histone lactylation can mediate polarization of mouse bone marrow macrophages, and the modification level is parallel to the intracellular lactate level.^[38]^ Collectively, these studies position lactate-mediated epigenetic regulation, particularly histone lactylation, as a central node in ischemic stroke pathophysiology. Building on this framework, we hypothesized that lactate-induced H3K9la may target transcription factors critical for resolving neuroinflammation—with nuclear factor erythroid 2-related factor 2 (Nrf2).

Lactate has been established as an important signaling molecule; however, its effects on microglial polarization and the role of histone lactylation in this process under cerebral ischemia. Microglia (also known as BV2 cells) play a crucial role in regulating inflammation in ischemic stroke, serving as immune cells in the central nervous system. Their polarization state, particularly the balance between proinflammatory M1 and anti-inflammatory M2 phenotypes, directly influences neurological functional recovery. Therefore, studying the molecular mechanisms that regulate the polarization of microglia has both significant academic value and potential clinical translation. This study sought to investigate the mechanism by which lactate regulates Nrf2 and activates downstream NF-κB through histone lactylation modification, and elucidate the role of the H3K9la-Nrf2/NF-κB axis in inflammation. The research results will provide new ideas and treatment targets for the study and treatment of this patient population.

## Materials and methods

### Cell culture and treatments

BV-2, SH-SY5Y, HT22 cell lines, and primary mouse microglia (CP-M110) (Procell, China) were cultured in Dulbecco’s Modified Eagle Medium (DMEM; Gibco 11965092) supplemented with 10% fetal bovine serum (FBS; Gibco A5256701) and 1% penicillin-streptomycin (Gibco 15140122) at 37 °C in a 5% CO_2_ atmosphere, following the supplier’s protocols. The cells were exposed to sodium lactate (Sigma L7022) at concentrations ranging from 0 to 50 mmol/ L for 24 to 48 h, lipopolysaccharide (LPS; Sigma L4391) for 6 to 24 h, or C646/SnPPIX (Selleck S7152/S7879) with a dimethyl sulfoxide (DMSO) concentration of ≤ 0.1%. Cell viability was evaluated using the Cell Counting Kit-8 (CCK-8; Beyotime C0038) by incubating 1×10^4^ cells per well in 96-well plates with 10% CCK-8 solution for 2 h. Absorbance was subsequently measured at 450 nm using a SpectraMax i3x spectrophotometer.

### Middle cerebral artery occlusion (MCAO) modeling

Adult male C57 mice, aged 8 weeks and weighing 22 ± 2 grams, were anesthetized with 3% isoflurane and maintained at 1.5% *via* endotracheal intubation. Transient focal cerebral ischemia was induced by inserting a silicone-coated 6–0 monofilament, measuring 11.0 ± 0.5 mm, into the right internal carotid artery to occlude the origin of the middle cerebral artery. Tissue collection was performed at 4, 8, and 12 h post-ischemia. All animal procedures were conducted in accordance with the guidelines of the Animal Care and Use Committee of Jilin University (Approval No. JDYY-2024–0372).

The mice were divided into control group, MCAO group, and MCAO+LA group. MCAO+LA group mice were injected with lactate into the cerebral cavity after cerebral ischemia, while the remaining groups were injected with physiological saline. Each group of experimental animals were placed in an open field and conduct behavioral evaluations on the mice to assess their neurological damage through Grip Strength test, Rotarod test, and Corner test.^[39, 40, 41]^

### Quantitative RT-PCR (qRT-PCR)

Total RNA was extracted utilizing TRIzol (Invitrogen, 15596026CN). The purity and concentration of the RNA were assessed using a NanoDrop 2000 spectrophotometer. First-strand cDNA synthesis was conducted using reverse transcriptase (Vazyme, R323). Quantitative PCR was conducted with the ChamQ Universal SYBR qPCR Master Mix (Vazyme, Q321). Primer sequences are detailed in Supplementary Table 1. Gene expression levels were normalized to β-actin and analyzed using the 2^-ΔΔCt^ method.

### Western blotting

Cells were lysed using RIPA buffer (Thermo, 89900) supplemented with a protease inhibitor cocktail (Thermo, A32965) at 4 °C for 30 min. The lysates were subsequently subjected to centrifugation at 12,000 × *g* for 15 min at 4 °C to separate cellular debris. The protein concentrations of the resulting supernatants were quantified using a BCA assay (Thermo, A65453). Equal aliquots of protein (20 μg) were then separated by electrophoresis on 10% Bis-Tris gels (Beyotime, P0866M) at a constant voltage of 90 V for 2 h, followed by transfer to PVDF membranes (Millipore, IPVH00010) at 200 mA for 90 min. The membranes were blocked with 5% BSA (Sangon, A602440) in PBST for 1 h, after which they were incubated overnight at 4 °C with primary antibodies: anti-H3K9la (PTM Biolabs, PTM-1419RM; 1:1000), anti-Nrf2 (Cell Signaling Technology, 12721T; 1:500), anti-HO-1 (CST, 26416; 1:1000), anti-NF-κB p65 (CST, 8242; 1:500), antiphospho-NF-κB p65 (CST, 3033; 1:500), anti-IκBα (CST, 4812; 1: 500), anti-phospho-IκBα (CST, 9246; 1:500), and anti-β-actin (Proteintech, 66009–1; 1:5000). Following the primary antibody incubation, HRP-conjugated secondary antibodies (Proteintech, RGAR001/SA00001; 1:5000) were applied for 1 h prior to detection using ECL (CST, 6883). Quantification of the resulting signals was performed using Image Lab software (Bio-Rad).

### Cut& Tag analysis

CUT& Tag analysis was performed using a commercial kit (Vazyme, TD903) according to the manufacturer’s protocols. Briefly, 100, 000 sorted cells were immobilized on ConA magnetic beads, permeabilized with a buffer containing 0.01% digitonin, and sequentially incubated with primary antibodies against H3K9la (PTM Biolabs, PTM-1419RM; 1:50 dilution in antibody buffer, 2 h at room temperature) and secondary antibodies (30 min at room temperature). Following two washes with digitonin wash buffer to eliminate unbound antibodies, tagmentation was performed using activated Tn5 transposase (37 °C, 60 min). DNA fragments were purified using Vazyme DNA Clean Beads, and libraries were constructed through 15-cycle PCR amplification with indexed primers.

### Chromatin immunoprecipitation (ChIP) assay

ChIP assays were conducted utilizing the Chromatin IP Kit (Cell Signaling Technology, CST 9004), employing 4 × 10^6^ cells per immunoprecipitation. Cells were crosslinked with formaldehyde for 10 min at room temperature and subsequently quenched with glycine. Chromatin was enzymatically fragmented using micrococcal nuclease to produce fragments ranging from one to five nucleosomes. The digested chromatin was incubated overnight at 4 °C with 5 μg of anti-H3K9la antibody (PTM Biolabs, PTM-1419RM) or Nrf2 polyclonal antibody (Proteintech, 16396–1-AP) and normal rabbit IgG (CST, 2729) immobilized on protein G agarose beads. Following a series of washes, crosslinks were reversed, and DNA was purified. Enrichment of the Nrf2 or Arg1 promoter were quantified using qPCR with gene-specific primers, normalized to IgG controls and input samples, and calculated using the 2^-ΔΔCt^ method.

### Enzyme-linked immunosorbent assay (ELISA)

Cytokine concentrations in cell supernatants (centrifuged at 1000×*g* for 10 min), and brain injury tissue protein (centrifuged at 12,000×*g* for 15 min) were determined using ELISA kits specific for tumor necrosis factor-alpha (TNF-α) (Thermo, BMS607), IL-6 (Thermo, KMC0061), and IL-1β (Beyotime, PI301), following the protocols provided by the manufacturers.

### Flow cytometry

Cells were harvested, washed with phosphate-buffered saline (PBS), and stained with anti-CD206 (Invitrogen, 53–2061–82), anti-CD86 (Invitrogen, 11–0862–82), and anti-CD11b (Cell Signaling Technology, 41249) antibodies for 30 min at 4 °C. The samples were analyzed using a Beckman CytoFLEX LX flow cytometer, and the data were processed with FlowJo V10 software.

### TTC staining

Brain infarction was assessed using 2, 3, 5-triphenyltetrazolium chloride (TTC) staining (Beyotime, C0651). Coronal sections (2 mm) from MCAO model brains were incubated in 2% TTC saline solution at 37 °C for 15 min at 80 rpm in the dark, followed by fixation in 4% paraformaldehyde (PFA) overnight. Viable tissue was stained deep red, while infarcted areas remained unstained (white). Infarct volume was quantified relative to the total volume of the ipsilateral hemisphere through blinded analysis using ImageJ software.

### TUNEL assay

Apoptosis was evaluated using the TUNEL assay (Beyotime, C1089). Formalin-fixed, paraffin-embedded (FFPE) sections were deparaffinized, rehydrated, and digested with Proteinase K (20 μg/mL) at 37 °C for 30 min. After washing in PBS, the sections were equilibrated in TUNEL Dilution Buffer and incubated with the TUNEL Reaction Mixture at 37 °C for 60 min in a dark, humid chamber. Following PBS washes and counterstaining with DAPI (1 μg/mL), the slides were mounted and analyzed using fluorescence microscopy.

### Immunofluorescence

MCAO mice were euthanized at 4, 8, and 12 h after ischemia induction. The brain tissue will be sliced into 10 μm coronal sections and subjected to immunofluorescence staining using primary antibodies against Iba1 (pan microglial cell marker, Abcam, ab178847), M1 marker CD86 (Invitrogen, 11–0862–82), and M2 marker CD206 (Invitrogen, 53–2061–82). The secondary antibody combined with Alexa Fluor 488 (for Iba1) and Alexa Fluor 594 (for CD86/CD206) will be used for signal detection. Capture images using a confocal laser microscope (Zeiss LSM 900). The region of interest (ROI) was selected from the ischemic core and penumbra (defined by TTC staining and anatomical markers).

### Hematoxylin-Eosin (HE) staining

We taked brain coronal sections (2 mm thick) stained with TTC and fixed overnight with 4% PFA, and performed gradient dehydration with ethanol. The slices were embedded in paraffin and continuous paraffin sections with a thickness of 5 μm were made. After the steps of dewaxing to water, hematoxylin staining, differentiation and blue-green staining, eosin staining, and dehydration transparency, neutral gum sealing is performed. An optical microscope (Zeiss Axio Imager Z2) was used to capture images of the ischemic core area, penumbra, and contralateral normal area under a 40× objective lens.

### Nissl staining

Coronal sections of brain were taken from the ischemic (ipsilateral) and intact (contralateral) hemispheres, focusing on the cortex and striatum (regions most affected by MCAO. Brain sections underwent deparaffinization followed by staining with 0.1% cresyl violet (Beyotime C0117; 40 °C for 10 min). Subsequently, sections were differentiated in ethanol gradients and cleared. The optical fractionator method (a standard stereological technique for unbiased cell counting) was used to define 5 random ROIs per section under a 20× objective. Only neurons with intact Nissl bodies (clear cytoplasmic staining) and visible nuclei were counted, excluding glia and damaged neurons. Neuronal densities in the infarcted (ipsilateral) and intact (contralateral) hemispheres were quantified from bright-field images using ImageJ software.

### Lactate quantification

Samples were homogenized in cold PBS at a ratio of 1: 10 (w/v), deproteinized with 8% perchloric acid, and neutralized using potassium hydroxide (KOH). Lactate concentrations were quantified utilizing a Lactate Assay Kit (Sigma MAK064). A 50 μL sample was mixed with 50 μL of Lactate Assay Reagent (LDH/NAD^+^) and incubated for 30 min at 25 °C. Absorbance was subsequently measured at 570 nm.

### Site-directed Mutagenesis of H3K9 to H3R

The H3K9R mutant plasmid group and the empty vector control group were both purchased from the Nanjing Public Protein/Plasmid Library (PPL). Incubate the plasmid with transfection reagent (Lipofectamine 3000) and add it dropwise to 293T cells for further cultivation. Collect cells after transfection and validate H3K9R protein expression through Western blot to confirm transfection efficiency.

### Statistical analysis

Data were presented as mean ± standard deviation (SD). Statistical analyses were conducted using GraphPad Prism version 10. Unpaired two-tailed Student’s *t*-tests were employed for comparisons between two samples, while one-way analysis of variance (ANOVA) was utilized for comparisons among multiple groups.

## Results

### Lactate enhances protein lactylation and induces M2 polarization in BV-2 cells

**Figure 1:**
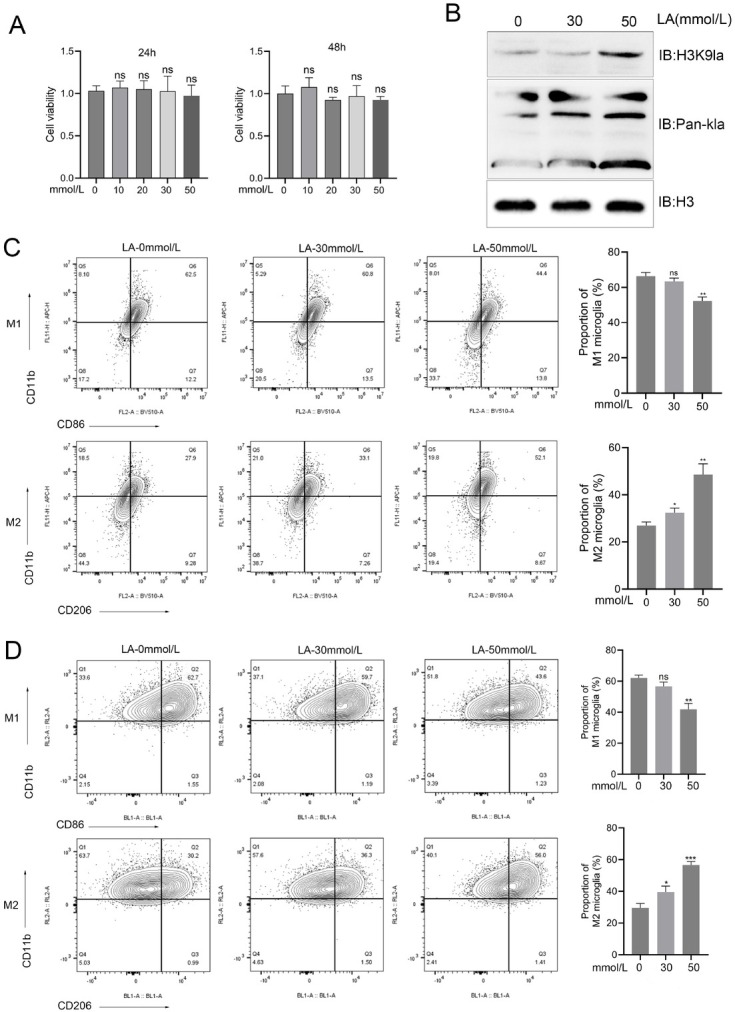
Lactate induces protein lactylation and promotes M2 polarization in BV-2 microglia. (A) Viability of BV-2 cells treated with 0-50mM (mmol/L) lactate for 24 h or 48 h assessed by CCK-8 assay (ns *vs*. control). (B) Western blot analysis of pan-lysine lactylation (pan-Kla) and H3K9la in cells treated with lactate (0, 30, 50 mmol/L) for 24 h. (C-D) Flow cytometry analysis of M1/M2 polarization markers in BV2 cells (C) and primary microglia (D). Representative pseudocolor density plots illustrate the distribution of microglial phenotypes following lactate treatment. Quantitative analysis reveals significant reductions in M1 markers and elevations in M2 markers compared to the control (Student’s *t*-test; mean ± SD, *n* = 3). ^*^P < 0.05, ^**^P < 0.01. CCK-8: Cell Counting Kit-8. ns, not significant; ^***^P < 0.001.

To evaluate the potential cytotoxic effects of lactate, the viability of BV-2 cells was assessed using the CCK-8 assay after pretreatment for 24 or 48 h with various concentrations of lactate (ranging from 0 to 50 mmol/L). The results indicated that lactate concentrations of 10 mmol/L, 20 mmol/L, 30 mmol/L, and 50 mmol/L did not significantly affect BV-2 cell viability when compared to the untreated control group (0 mmol/ L) at both time points (Figure 1A). Considering previous studies suggesting that lactate may enhance protein lactylation, we further examined its impact on global and histone lactylation within BV-2 cells. Western blot analysis demonstrated that treatment with 30 mmol/L and 50 mmol/L lactate for 24 h significantly elevated the levels of pan-Kla (pan-lysine lactylation) and the specific histone modification H3K9la (histone H3 lysine 9 lactylation) relative to untreated cells (0 mmol/ L) (Figure 1B).

Next, to determine the impact of lactate exposure on BV-2 cell and primary microglia polarization, flow cytometry was conducted to assess macrophage polarization markers after a 24-hour treatment with lactate concentrations of 0 mmol/L, 30 mmol/L, or 50 mmol/L. The results demonstrated that lactate exposure significantly altered the polarization state, as indicated by a marked decrease in markers associated with the pro-inflammatory M1 phenotype. Concurrently, there was a notable increase in markers indicative of the anti-inflammatory M2 phenotype in lactate-treated cells (Figure 1C-D).

### Lactate-induced modulation of microglial polarization towards the M2 phenotype in ischemic brain

**Figure 2:**
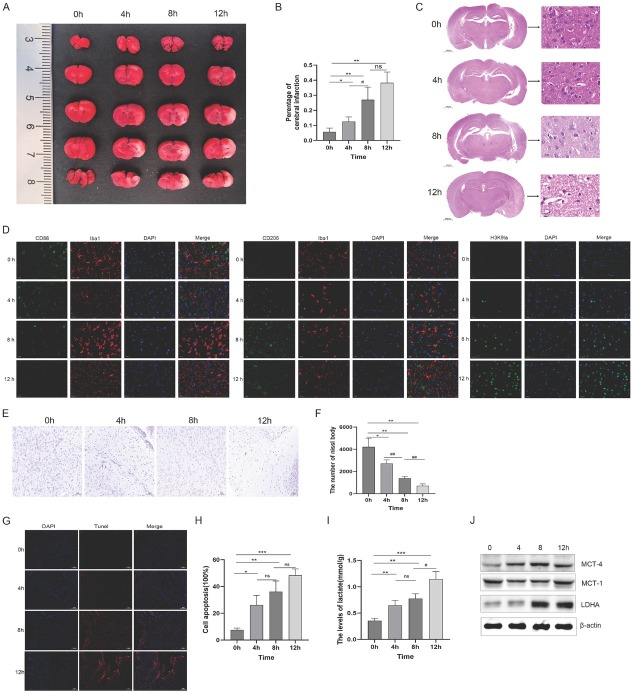
Temporal multimodal profiling of ischemia progression in transient MCAO mice. (A) Cerebral infarction visualization: Serial 1-mm coronal sections stained with 2% TTC after 37 °C incubation for 20 min. White regions demarcate infarction zones. (B) Volumetric quantification: Infarct volumes calculated using ImageJ software. Data presentation: group mean values with SEM error bars (*n* = 5 mice/group). (C) HE staining within 0-12 h after cerebral ischemia. (D) Immunohistochemical staining after cerebral ischemia. (E) Representative images of Nissl-stained brain sections (scale bar: 100 μm). (F) Quantification of Nissl bodies staining. (G) Typical fluorescence photomicrograph of TUNEL staining (scale bar: 200 μm). (H) Quantitative analysis of TUNEL-positive cells. (I) Lactate concentration in ischemic brain tissue was measured using a lactate assay kit. (J) Expression of MCT1, MCT4, LDHA, and β-actin protein in ischemic brain tissue was analyzed by Western blotting. MCAO: middle cerebral artery occlusion; TTC: 2, 3, 5-triphenyltetrazolium chloride. ns, not significant; ^*^P < 0.05; ^#^*P* < 0.05; ^**^P < 0.01; ^##^*P* < 0.01; ^***^P < 0.001.

Building on the observed capacity of lactate to induce protein lactylation and facilitate the transition to an anti-inflammatory M2 phenotype in BV-2 microglia *in vitro* (Figure 1), we next examined the temporal dynamics of lactate metabolism and microglial polarization within the context of cerebral ischemia *in vivo*. Transient MCAO in mice revealed three distinct pathological phases within the 4–12-hour post-ischemia interval. TTC staining demonstrated a significant increase in infarct volume (% ipsilateral hemisphere) from 12.5% ± 3.0% at 4 h to 27.0% ± 8.5% at 8 h (*P* < 0.05), with no further progression at 12 h (38.2% ± 7.0%, not significant)(Figure 2A and B). HE staining shows that the cerebral cortex (CTX), hippocampus (HP), caudate putamen (CP), and thalamus (TH) all produce a large number of vacuoles and nuclear contractions, and their intact tissue structures are damaged after cerebral ischemia for 4–12 h. And as the ischemic time increases, the brain tissue damage becomes more severe (Figure 2C). Similar to Figure 1C& D, the positive signals of Iba1^+^ CD206^+^ (M2) microglia, and the positive signals of H3K9la^+^ microglia significantly increased, while the positive signals of Iba1^+^ CD86^+^ (M1) microglia significantly decreased after lactate exposure (Figure 2D). In contrast, Nissl staining revealed progressive neuronal loss as evidenced by a decline in viable neuron density from 4214 ± 772 cells/mm^2^ at 0 h to 2719 ± 331 cells/mm^2^ at 4 h (*P* < 0.05 *vs*. 0 h), to 1385 ± 155 cells/mm^2^ at 8 h (*P* < 0.01 *vs*. 4 h), and finally to 709 ± 171 cells/mm^2^ at 12 h (*P* < 0.01 *vs*. 8 h)(Figure 2E-F). Concurrently, TUNEL staining revealed progressive apoptotic neuronal death, with rates significantly increasing from 7.5% ± 1.4% at 0 h to 26.1% ± 7.3% at 4 h (*P* < 0.05 *vs*. 0 h), 36.2% ± 8.0% at 8 h, and peaking at 48.4% ± 4.7% at 12 h (Figure 2G-H). Western blot analysis revealed time-dependent upregulation of lactate transporters. Elevated protein levels of MCT1, MCT4, and LDHA were observed in ischemic samples between 4–12 h post-occlusion compared to controls, suggesting enhanced activation of the lactate transport system (Figure 2J).

To evaluate the translational value of lactate in functional recovery, we performed behavioral tests on three experimental groups (Control group, MCAO group, MCAO+LA group). The behavior of mice was evaluated using Grip Strength test, Rotarod test, and Corner test (Supplementary Figure S1). The MCAO group showed a significant increase in grip strength in mice, which compared with the control group (*P <* 0.01). The MCAO+LA group showed a certain decrease in grip strength, which compared with the MCAO group (*P <* 0.05). Similarly, the LA treatment group also showed better motor function recovery characteristics compared to the MCAO group in the Rotarod test, and Corner test (Supplementary Figure S1B-D). This result confirms that lactate reduces neurological impairment in MCAO mice.

**Figure 3:**
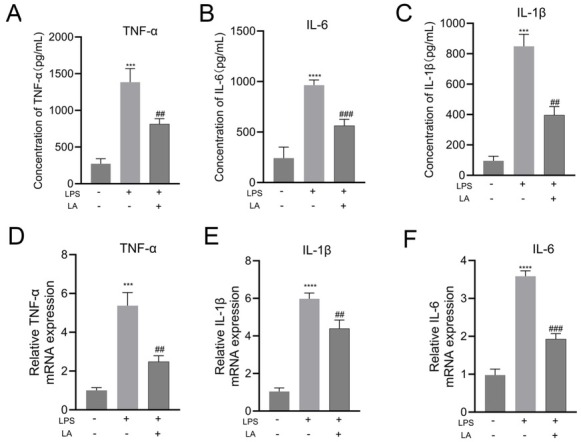
Effects of lactate on the production of pro-inflammatory cytokines in LPS-induced BV-2 cells. After pretreatment with different concentrations of lactate (LA) for 1 h, BV-2 cells were treated with 1 μg/mL LPS for 24 h or 12 h. The protein levels of TNF-α (A), IL-6 (B) and IL-1β (C) were measured using commercial ELISA kits. The mRNA levels of TNF-α (D), IL-6 (E), and IL-1β (F) were determined by real-time PCR. All experiments were repeated at least three times, and similar results were observed. ^***^P < 0.001, ^****^P < 0.0001 *vs*. the control group. ^##^*P* < 0.01, ^###^*P* < 0.001 *vs*. the LPS-treated group.

Complementary *in vitro* experiments demonstrated that lactate exerted direct anti-inflammatory effects on BV-2 microglia. Specifically, pretreatment with 50 mmol/L lactate significantly suppressed LPS-induced cytokine production, reducing TNF-α from 1383.7 ± 185.6 to 814.0 ± 71.0 ng/L (*P* < 0.01), IL-6 from 964.3 ± 50.8 to 562.7 ± 62.7 ng/L (*P* < 0.001), and IL-1β from 848.3 ± 79.7 to 396.3 ± 56.0 ng/L (*P* < 0.01) compared to LPS-only controls (Figure 3A-C). At the transcriptional level, quantitative PCR analysis showed significant reductions in LPS-induced mRNA expression in lactate-treated cells, with TNF-α decreased by 2.2-fold (*P* < 0.01), IL-6 by 1.9-fold (*P* < 0.01), and IL-1β by 1.4-fold (*P* < 0.01) compared to LPS-only controls (Figure 3D-F). In addition, similar to the results, these inflammatory factors (TNF-α, IL-6, IL-1β) were significantly elevated in ischemic brain tissue and primary microglia after LPS stimulation. However, lactic acid can reduce the expression of TNF-α, IL-6, IL-1β (Supplementary Figure S2 and S3). Collectively, these findings reveal a critical ischemic time window (4–12 h post-MCAO) during which metabolic reprogramming occurs in parallel with progressive neuronal damage while promoting an anti-inflammatory phenotypic shift in microglia.

### Epigenetic rewiring through H3K9 lactylation activates Nrf2-mediated cytoprotection

**Figure 4:**
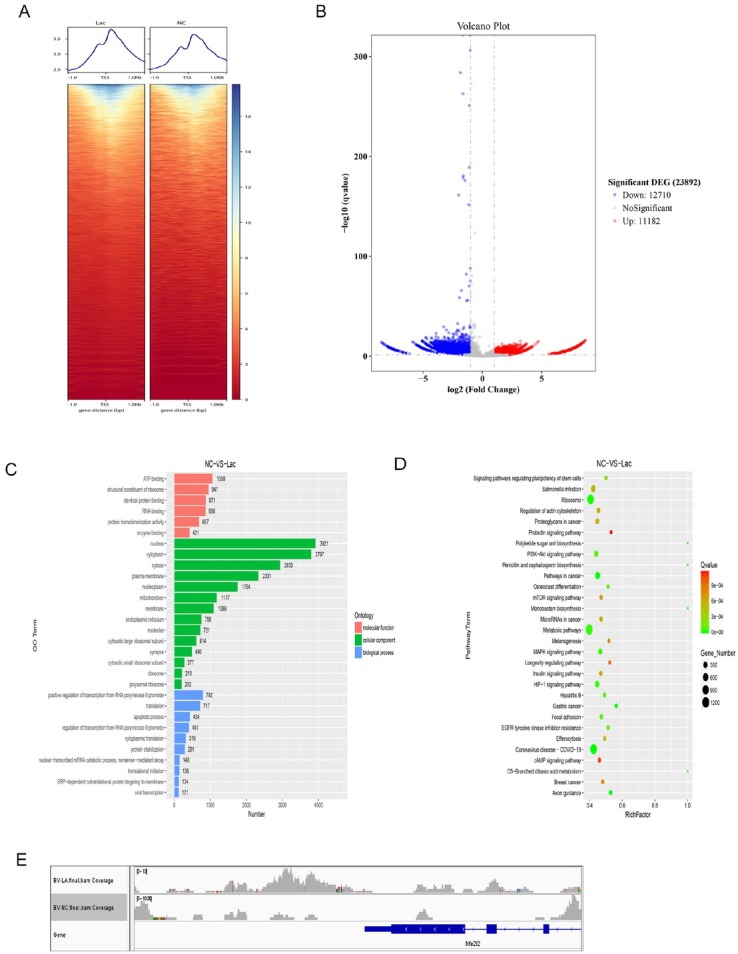
Genome-wide profiling of H3K9 lactylation in BV-2 microglia. (A) TSS-centered (±1 kb) chromatin density analysis. Heatmap visualization of CUT& Tag read distribution aligned to transcription start sites using species-specific GFF annotations. (B) Differential H3K9la peak identification. Volcano plot showing significantly altered peaks (| log_2_FC| ≥2, q ≤ 0.05) with upregulated (red) and downregulated (blue) loci. (C) Functional annotation of peak-associated genes. The top 30 enriched GO terms across biological processes, cellular components, and molecular functions are displayed as bar plots. (D) Pathway enrichment analysis. Scatter plot of top 30 KEGG pathways ranked by Rich factor (ratio of observed/expected genes) and significance (q-value). (E) Epigenomic landscape visualization. Multi-scale IGV browser tracks displaying read abundance across the Nrf2 genomic locus.

After establishing lactate-induced H3K9 lactylation and M2 polarization in BV-2 microglia, H3K9la-targeted CUT and Tag sequencing was conducted to elucidate the epigenetic landscape associated with these phenotypic alterations. Treatment with lactate (50 mmol/ L, 24 h) resulted in significant enrichment of H3K9la at transcriptional start sites (±2 kb TSS regions) (Figure 4A). Differential peak analysis identified 23, 892 loci with altered lactylation, including key anti-inflammatory regulators such as Nrf2 and Arg1, which demonstrated over a 3.5-fold increase in H3K9la at their promoters (Figure 4B). Functional annotation indicated that these targets were significantly overrepresented in M2 polarization pathways and the Nrf2-mediated oxidative stress response (Figure 4C-D). Multi-scale visualization using the IGV browser further confirmed the accumulation of lactate-induced H3K9la at the Nrf2 promoter, precisely overlapping with antioxidant response elements (Figure 4E). Taken together, these findings elucidate the mechanistic linkage between lactate-induced histone lactylation and the transcriptional activation of cytoprotective programs, thereby providing chromatin-level validation for the observed microglial repolarization (Figure 2I) and cytokine suppression (Figure 3D-F).

### H3K9 lactylation facilitates activation of the Nrf2/HO-1 antioxidant pathway in microglia

**Figure 5:**
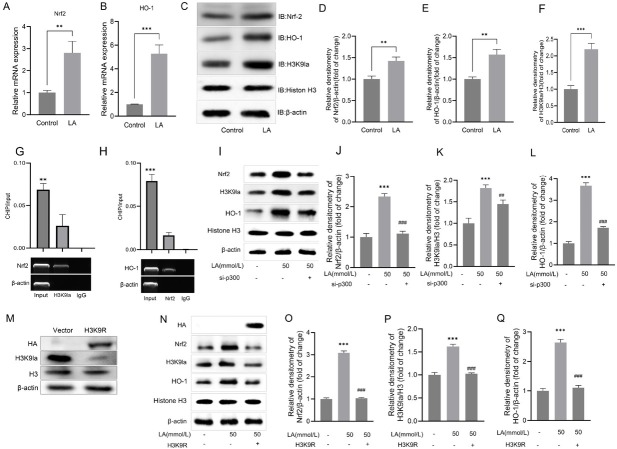
Lactate-induced H3K9 lactylation epigenetically activates Nrf2/HO-1 signaling in microglia. (A-B) qPCR analysis of Nrf2 and downstream effector HO-1 mRNA expression in lactate-treated BV-2 microglia. (C) Immunoblot detection and quantification of H3K9la, Nrf2, and HO-1 protein levels. (D-F) Quantitative data represent grayscale intensity measurements normalized to loading controls (mean ± SD, *n* = 3). (G) ChIP-qPCR validation of H3K9la enrichment at Nrf2 promoter coordinates. (H) ChIP-qPCR validates Nrf2 binding to the region of the HO-1 promoter. (I-L) Role of p300 in lactate-induced Nrf2/HO-1 activation:(I) Immunoblot detection of Nrf2, H3K9la, HO-1, Histone H3, and β-actin in BV-2 cells treated with lactate (50 mmol/L) and/or transfected with p300 siRNA (si-p300); (J-L) Quantitative analysis of relative densitometry for Nrf2, H3K9la, and HO-1, respectively, normalized to loading controls (mean ± SD, *n* = 3, ^***^P < 0.001 *vs*. control group, ^###^*P* < 0.001 *vs*. lactate group). (M) Immunoblot analysis showing the expression of H3K9R mutant and wild-type H3 in 293T cells transfected with corresponding plasmids, with β-actin as a loading control. (N-Q) Effects of H3K9R mutation on Nrf2/HO-1 signaling:(N) Immunoblot detection of HA-tagged H3K9R, Nrf2, HO-1, and histone H3 in cells treated with lactate and transfected with H3K9R or vector; (O-Q) Quantitative analysis of relative densitometry for Nrf2, HO-1, and H3K9la, respectively, normalized to loading controls (mean ± SD, *n* = 3, ^***^P < 0.001). ^**^P < 0.01; ^##^*P* < 0.01.

To elucidate the functional significance of H3K9 lactylation dynamics at the Nrf2 locus, we conducted a systematic investigation into its regulatory role in antioxidant signaling following genome-wide analysis. Exposure to lactate (50 mmol/ L, 24 h) resulted in a significant transcriptional activation of Nrf2, with a 2.8-fold increase (*P* < 0.01; Figure 5A), which subsequently led to a 5.3-fold induction (*P* < 0.001) of its canonical target, HO-1 (Figure 5B). Besides, immunoblotting analyses revealed an increase in H3K9la (2.2-fold), Nrf2 (1.4-fold), and HO-1 (1.6-fold) protein levels (all *P* < 0.01; Figure 5C-F). Locus-specific ChIP-qPCR quantification confirmed the enrichment of H3K9la (*P* < 0.01) within the ARE region of the Nrf2 promoter region (Figure 5G). Importantly, locus-specific ChIP-qPCR quantification confirmed enrichment of HO-1 (*P* < 0.001) within the Nrf2 promoter region (Figure 5H), directly supporting the spatial specificity predicted by CUT& Tag analysis in Figure 4E.

To further validate whether H3K9 is the critical site mediating lactate-induced Nrf2 activation, we constructed the H3K9 arginine mutant (H3K9R; Figure 5M). Experimental results showed that compared with the control group, the lactate-induced expression levels of Nrf2 and its downstream target gene HO-1 were significantly inhibited in cells transfected with H3K9R mutant. This result directly confirms that the H3K9 site is essential for lactate to regulate Nrf2 protein expression, further supporting the core conclusion that H3K9 lactylation (H3K9la) drives Nrf2 activation. These comprehensive analyses delineate a mechanistic pathway where lactate-induced H3K9 lactylation epigenetically primes the Nrf2/HO-1 antioxidant axis, thereby providing a molecular basis for the observed M2 polarization and ischemic neuroprotection.

### Lactate activates Nrf2/HO-1 signaling to attenuate NF-κB-mediated inflammatory responses in microglia

**Figure 6:**
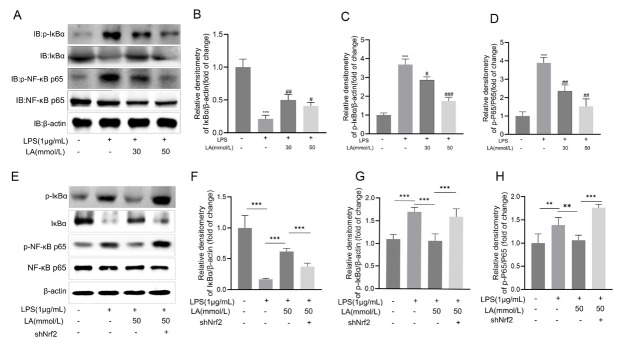
Lactate inhibits NF-κB activation in LPS-stimulated BV-2 microglia. BV-2 cells were pretreated with lactate (30 mmol/L and 50 mmol/L) for 1 h, followed by LPS (1 μg/mL) stimulation. (A) Western blot analysis of IκBα, NF-κB p65, and their phosphorylated forms. (B-C) IκBα and phospho-IκBα levels normalized to β-actin. (D) Quantification of phospho-NF-κB p65 levels normalized to total NF-κB p65. (E) Immunoblot detection of p-IκBα, IκBα, p-NF-κB p65, NF-κB p65, and β-actin in BV-2 cells treated with LPS (1 μg/mL), lactate (50 mM), and/or Nrf2 shRNA (shNrf) ;(F-H) Quantitative analysis of relative densitometry for IκBα, p-IκBα, and p-NF-κB p65, respectively, normalized to loading controls (mean ± SD, *n* = 3, ^**^P < 0.01, ^***^P < 0.001 *vs*. control group, ^###^*P* < 0.001 *vs*. LPS group, comparisons between indicated groups are marked by lines). ^#^*P* < 0.05; ^##^*P* < 0.01.

Pretreatment of BV-2 microglial cells with 30 mmol/ L or 50 mmol/L lactate for one hour prior to stimulation with LPS at a concentration of 1 μg/mL significantly attenuated the activation of NF-κB *via* the Nrf2/HO-1 signaling pathway. The activation of the NF-κB pathway upregulates pro-inflammatory cytokine production, with IκB-α acting as a critical inhibitor. To evaluate lactate’s effects on NF-κB signaling in LPS-stimulated cells, cultures were pretreated with lactate (30 mmol/ L, 50 mmol/ L) for 1 h prior to LPS exposure (1 h). Results demonstrated that lactate significantly inhibited LPS-induced IκB-α phosphorylation/degradation (Figure 6A-C) and potently suppressed phosphorylation of NF-κB p65 (Figure 6D). To mechanistically define Nrf2 as the pivotal mediator of lactate’s NF-κB inhibitory effect, we performed a genetic rescue experiment (Figure 6E-H): Nrf2 was knocked down in BV-2 cells using short hairpin RNA (shNrf2). Subsequently, Western Blot (WB) was utilized to detect the expression levels of key molecules in the NF-κB pathway, including phosphorylated IκBα (p-IκBα), total IκBα, phosphorylated NF-κB p65 (p-NF-κB p65), and total NF-κB p65. Experimental results showed that when Nrf2 was knocked down (shNrf2 group), the inhibitory effect of lactate on p-IκBα and p-NF-κB p65 was significantly weakened, and concurrently, the enhancing effect of lactate on total IκBα was also abrogated. This result directly proves that Nrf2 is an essential factor for lactate-mediated inhibitory effect on the NF-κB pathway, clearly establishing the regulatory relationship between them.

These collective findings establish that lactate’s anti-inflammatory mechanism is mediated through the suppression of NF-κB activation *via* blockade of IκBα degradation and p65 phosphorylation, and this process is critically dependent on Nrf2 signaling.

### Lactate suppresses pro-inflammatory cytokine expression via the Nrf2/HO-1 axis

Lactate, at a concentration of 50 mmol/L, significantly mitigated inflammation in LPS-stimulated BV2 microglia through the Nrf2/HO-1 axis, primarily *via* H3K9 lactylation-mediated epigenetic priming of Nrf2 transcription (Figures 5–6).

**Figure 7:**
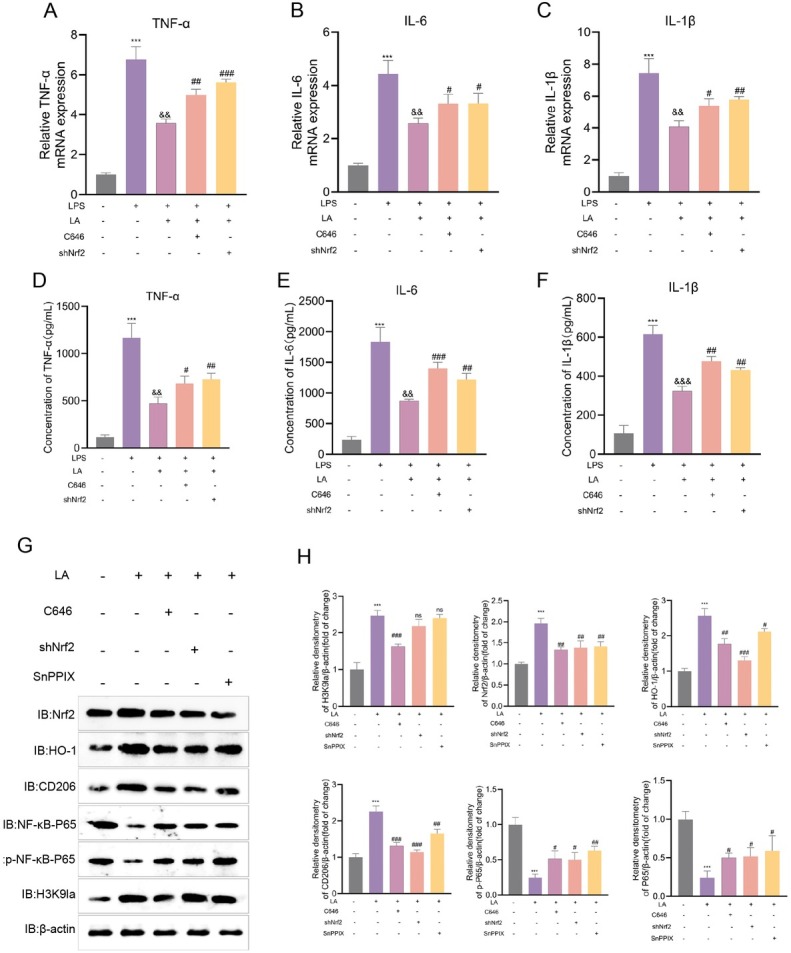
Lactate modulates microglial polarization through Nrf2/HO-1/NF-κB pathways. (A-C) BV-2 microglia were pretreated with C646 (10 μmol/L, 3 h), SnPP IX (40 μmol/L, 3 h) and lactate (50 mmol/L, 1 h) prior to LPS stimulation (12 h). mRNA expression levels of TNF-α, IL-6 and IL-1β were analyzed by RT-qPCR. (D-F) Secreted cytokine levels of TNF-α, IL-6 and IL-1β were measured by ELISA in cell culture supernatants. Data represents SD from three independent experiments. ^***^P < 0.001 *vs*. untreated control group; ^$$^*P* < 0.01, ^$$$^*P* < 0.001 *vs*. LPS-treated group; *P* < 0.05, *P* < 0.01, < 0.001 *vs*. LPS + lactate group. (G-H) Immunoblot analysis of Nrf2, HO-1, H3K9la, phospho-NF-κB p65, and CD206 protein levels in LPS-stimulated BV2 microglia. Cells were treated with 50 mmol/L lactate (LA) alone or in combination with: p300 inhibitor C646, Nrf2-targeting shRNA (shNrf2), or HO-1 inhibitor SnPP. ^***^P < 0.001 *vs*. LPS group; < 0.05, *P* < 0.01, *P* < 0.01 *vs*. LA-treated group. ns, not significant; ^#^*P* < 0.05; ^##^*P* < 0.01; ^###^*P* < 0.001.

This process resulted in a 42.1%–47.3% reduction in the mRNA expression of pro-inflammatory cytokines TNF-α, IL-6, and IL-1β (*P* < 0.01 compared to LPS; Figures 7A-C) and a 47.5%–59.4% decrease in cytokine secretion (*P* < 0.01; Figure 7D-F). Mechanistic investigations revealed a hierarchical regulation pathway: genetic knockdown of *Nrf2* (shNrf2) significantly reversed the immunosuppressive effects of lactate, restoring cytokine mRNA levels to 75.6%–83.4% and protein secretion to 62.3%–70.1% of LPS-induced levels (*P* < 0.001 compared to the lactate group). Pharmacological inhibition of p300 using C646 negated lactate’s effects, reinstating inflammatory mRNA responses to 72.6%–75.2% and protein responses to 58.4%–77.4% of LPS control levels (*P* < 0.01).

Under LPS stimulation, lactate treatment significantly activated the Nrf2/HO-1 signaling pathway while concurrently inhibiting NF-κB signaling. This was evidenced by a substantial increase in Nrf2 (2.0-fold compared to LA, *P* < 0.001), HO-1 (2.6-fold, *P* < 0.001), and H3K9 lactylation (2.5-fold, *P* < 0.001), alongside a reduction in phosphorylated NF-κB p65 (p-p65; 76% of LA, *P* < 0.001) and an increase in the M2 marker CD206 (2.3-fold, *P* < 0.001). Importantly, all interventions reversed the effects of lactate as evidenced by inhibition of p300 by C646, which eliminated H3K9 lactylation (1.5-fold) and suppressed the induction of Nrf2/HO-1 (1.4-fold), restoring p65 to 52% of LA levels. Knockdown of Nrf2 (shNrf2) markedly reduced HO-1 expression (0.5-fold) and negated lactate’s immunosuppressive effects (p-p65: 2.0-fold). Furthermore, inhibition of HO-1 activity by SnPPIX abolished lactate-induced CD206 expression (0.27-fold compared to LA, *P* < 0.01) and partially decreased HO-1 protein levels (0.18-fold compared to LA) (Figure 7G-H). These findings collectively suggest that lactate exerts anti-inflammatory effects by activating the Nrf2/HO-1 axis through p300-mediated H3K9 lactylation, which in turn suppresses NF-κB p65 phosphorylation and significantly reduces the expression of pro-inflammatory cytokines in LPS-stimulated microglia.

### The role of lactate in mitigating microglia-induced neurotoxicity in SHSY5Y and HT22 cells

**Figure 8:**
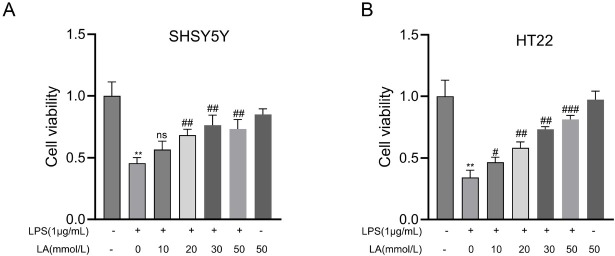
Lactate mediates neuroprotective effects via microglial polarization. SH-SY5Y and HT22 neuronal cells were cultured for 24 h with conditioned medium from microglia pretreated with different lactate concentrations (0, 10, 20, 30, 50 mmol/L) in the presence or absence of LPS (1 μg/mL), followed by cell viability assessment using the CCK-8 assay. Data is presented as mean ± SD (*n* = 5 independent experiments). ^**^P < 0.01 *vs*. untreated control group; *P* < 0.01, < 0.001 *vs*. LPS-treated group. ns, not significant; ^#^*P* < 0.05; ^##^*P*< 0.01; ^###^*P* < 0.001.

Research has demonstrated that activated microglia can exert neurotoxic effects primarily through the excessive release of pro-inflammatory cytokines. This study aimed to investigate whether lactate could confer neuroprotective effects by inhibiting microglial activation. To this end, conditioned medium (CM) was harvested from BV-2 cells under three different conditions: untreated control, stimulation with LPS for 24 h, and LPS stimulation following pretreatment with lactate at various concentrations. The CM was subsequently administered to cultured SHSY5Y and HT22 neuronal cells for a 24-hour incubation period, after which neuronal viability was assessed using the CCK8 assay. The results indicated that lactate pretreatment significantly attenuated neuronal cell death in a concentration-dependent manner (Figure 8A and B). These findings collectively suggest that lactate can potentially mitigate neuroinflammation-induced neurotoxicity by attenuating the production of proinflammatory mediators.

## Discussion

The increasing incidence of ischemic stroke poses a significant challenge to global health, as it remains a leading cause of morbidity and mortality. Ischemic stroke occurs when there is a reduction in blood flow to the brain, leading to neuronal death and functional impairment.^[42]^ The pathological processes following ischemic stroke include inflammation, oxidative stress, and metabolic dysregulation, particularly involving lactate accumulation due to anaerobic glycolysis. In the context of ischemic stroke, understanding the roles of microglia, the central nervous system’s resident immune cells, is crucial, given their involvement in polarization between M1 and M2 phenotypes. M1 microglia contributes to neuroinflammation and tissue damage, while M2 microglia are involved in tissue repair and anti-inflammatory responses. Therefore, unraveling the mechanisms governing microglial polarization following an ischemic stroke may provide insights for therapeutic strategies aimed at mitigating brain injury and promoting recovery.^[43,44]^

In this study, the impact of lactate accumulation was investigated on microglial polarization and its downstream effects on the *Nrf2* signaling pathway and NF-κB activity. Our findings indicated that lactate promoted M2 polarization of microglia while inhibiting M1 activation through mechanisms involving histone modification and transcription factor regulation. Specifically, lactate-induced H3K9 lactylation enhanced *Nrf2* gene expression, which in turn upregulated antioxidant responses and reduced pro-inflammatory cytokine release *via* NF-κB inhibition. Although this study preliminarily confirmed the critical role of the H3K9 site in lactate-mediated regulation of the Nrf2 pathway using exogenous mutant plasmids, the use of endogenous gene editing technologies (*e.g*., CRISPR-Cas9) to establish stable mutant cell lines or animal models would help validate the specific function of H3K9 lactylation in a context closer to physiological conditions. This represents an important objective for our subsequent investigations. These results highlight the potential of lactate as a modulator of microglial behavior in ischemic stroke and establish a foundation for future research aimed at exploiting this metabolic pathway for therapeutic benefit.^[43, 44, 45, 46, 47]^

Interestingly, the results of this study reveal a significant molecular mechanism through which lactate accumulation influences the polarization of microglia in the context of ischemic stroke. Specifically, lactate-induced H3K9la modification facilitates the expression of the *Nrf2* gene, a critical regulator of antioxidant responses, thereby promoting the M2 phenotype of microglia while simultaneously suppressing the M1 phenotype. Overall, this finding provides compelling evidence that lactate serves not only as a metabolic byproduct but also as a signaling molecule that can modulate inflammatory responses within the central nervous system (CNS) during ischemic conditions. The implications of these results extend to the therapeutic landscape, suggesting that enhancing *Nrf2* activation and promoting M2 polarization could represent a viable strategy to mitigate neuroinflammation and promote neuroprotection following ischemic stroke, aligning with previous studies that highlighted the protective roles of *Nrf2* in various pathological conditions, including neurodegenerative diseases.^[16,44]^

Furthermore, our findings revealed that lactate could inhibit the NF-κB signaling pathway, emphasizing its potential to modulate inflammatory responses in microglia. NF-κB is a well-established transcription factor that regulates the expression of pro-inflammatory cytokines, and its activation is associated with M1 microglial polarization. By inhibiting this pathway, lactate may reduce the release of inflammatory mediators, thus providing a dual benefit of promoting neuroprotection through M2 polarization while limiting the deleterious effects of M1 activation. This observation is supported by literature suggesting that dysregulation of NF-κB signaling contributes to chronic inflammation and tissue damage in various CNS disorders.^[48,49]^ Overall, these findings highlight the need for further investigation into the therapeutic targeting of lactate signaling pathways as a means to enhance recovery and repair mechanisms following ischemic stroke.

In addition to the molecular mechanisms discussed, the role of Nrf2 in modulating gene expressions related to microglial polarization underscores the significance of this transcription factor as a key regulator of cellular responses to oxidative stress and inflammation. Our findings suggest that the upregulation of HO-1, a downstream target of Nrf2, is correlated with enhanced M2 polarization, which is associated with tissue repair and neuroprotection. This aligned with existing research that demonstrates the importance of Nrf2 in maintaining cellular homeostasis and counteracting inflammatory processes within the CNS.^[50,51]^ Thus, understanding the interplay between lactate metabolism, Nrf2 signaling, and microglial polarization not only advances our knowledge of stroke pathology but also opens new avenues for therapeutic interventions aimed at harnessing the innate regenerative capacity of the CNS. Future studies should explore the translational potential of these findings to develop targeted therapies that can effectively modulate microglial function and improve outcomes in patients suffering from ischemic stroke.

The limitations of this study primarily include the absence of *in vivo* validation of our study findings, which restricts the applicability of our results to clinical settings. Besides, the relatively small sample size may limit the statistical power and generalizability of the conclusions drawn regarding lactate’s role in modulating microglial polarization. Furthermore, the study lacks a comprehensive exploration of the clinical relevance of the observed molecular mechanisms, which could provide a more robust understanding of the translational potential of targeting lactate in the treatment of ischemic stroke. Elevated lactate levels are often observed in human ischemic stroke patients.^[52]^

In clinical practice, acute phase of ischemic stroke triggers a significant inflammatory response, and lactate accumulation resulting from focal ischemia is one of the key contributing factors. A study confirmed that lactate levels are significantly elevated in the ischemic core, and this elevation is closely associated with a reduction in the apparent diffusion coefficient (ADC) and prolongation of the mean transit time (MTT). These parameters are reliable indicators of tissue ischemia and perfusion injury, and such a damaged microenvironment is known to drive the polarization of microglia toward an activated state (M1/M2 phenotypes).^[53]^ Systemic inflammatory responses are consistent with this local inflammation: serum inflammatory markers (*e.g*., interleukin-18, IL-18) are significantly increased, which correlates with stroke severity and risk of mortality.^[54]^ TNF-α also influence individual inflammatory responses and susceptibility to severe inflammation post-stroke.^[55]^ Beyond specific cytokines, systemically measured inflammatory markers commonly used in clinical practice also exhibit prognostic value. For instance, indicators like C-reactive protein (CRP) show correlations with stroke outcomes.^[56]^

Some studies suggest that lactate may stimulate M2 like polarization of macrophages through the ERK/STAT3 signaling pathway.^[57]^ In addition, lactate levels in stroke patients are correlated with clinical outcomes. The sustained slow decline in serum lactate concentration is associated with an increased risk of death.^[58]^ Elevated lactate levels in cerebrospinal fluid (CSF) of stroke patients are negatively correlated with astrocyte mitochondria.^[32]^ Future investigations should aim to address these limitations by incorporating larger sample sizes and validating the findings in experimental models that closely mimic the human condition.

In conclusion, our research elucidates the pivotal role of lactate in modulating microglial polarization, providing insights into the underlying molecular mechanisms that govern this process. By demonstrating that lactate influences the expression of Nrf2 and HO-1 while inhibiting NF-κB signaling, we contribute to a deeper understanding of the immune responses in ischemic stroke. These findings not only enhance our knowledge of microglial function but also suggest potential therapeutic targets for mitigating neuroinflammation and promoting neuroprotection in clinical settings. Future studies should investigate the application of these insights in the development of innovative therapeutic strategies to enhance recovery following ischemic injury.

## Conclusion

**Figure 9:**
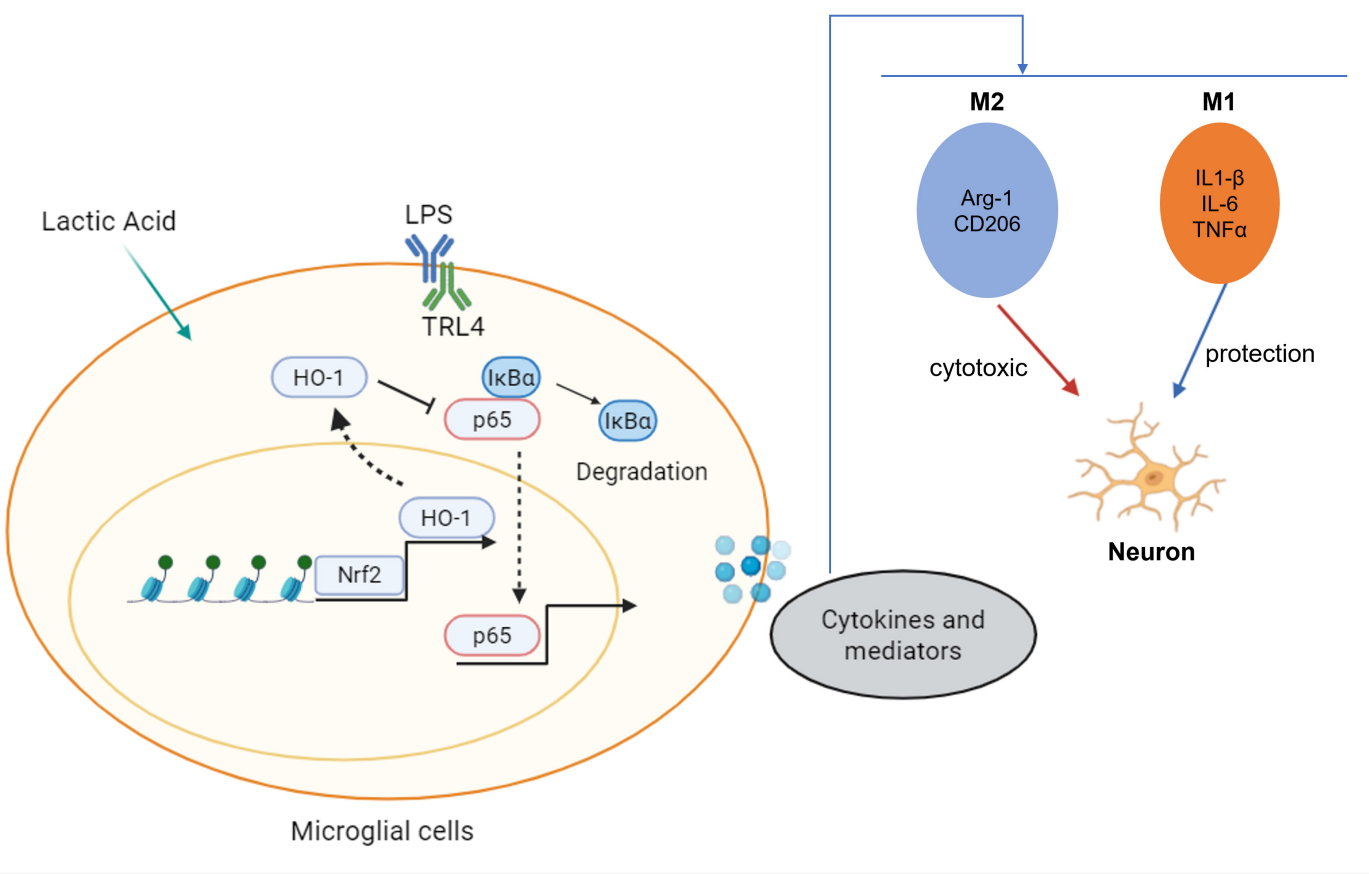
Lactate regulates microglia polarization and exerts neuroprotective effects *via* the H3K9la/Nrf2/HO-1-NF-κB signaling axis.

This study reveals a novel lactate/H3K9la/Nrf2/ HO-1/NF-κB signaling axis that reprograms microglial polarization in ischemic stroke. Specifically, lactate-derived H3K9 lactylation (H3K9la) epigenetically enhances Nrf2 transcriptional activity, driving HO-1 expression. HO-1 stabilizes IκBα, thereby reducing NF-κB phosphorylation and suppressing its transcriptional activation of inflammatory genes (*e.g*., TNF-α, IL-1β), which shifts microglia from a pro-inflammatory M1 to an anti-inflammatory M2 phenotype. Through this axis, lactate directly protects neurons from inflammatory injury (Figure 9) and mitigates brain damage *in vivo*. As an endogenous metabolite with dual epigenetic and immunomodulatory functions, lactate represents a promising therapeutic strategy against stroke-induced neuroinflammation. Future work will explore H3K9la dynamics in clinical stroke and optimize lactate delivery for translational applications.

## Supplementary Material

Supplementary Material Details
