# Supplementary material for: Lactate modulates microglial M2 polarization via H3K9 lactylation in ischemic stroke
Source: J Transl Int Med. 2026 Mar 26;14(2):276–93. doi: 10.1515/jtim-2026-0010 (PMC13110458; doi:10.1515/jtim-2026-0010)
Supplement: Supplementary file 1 — Supplementary Material Details [file jtim-2026-0010_sm.pdf]

Supplementary materials

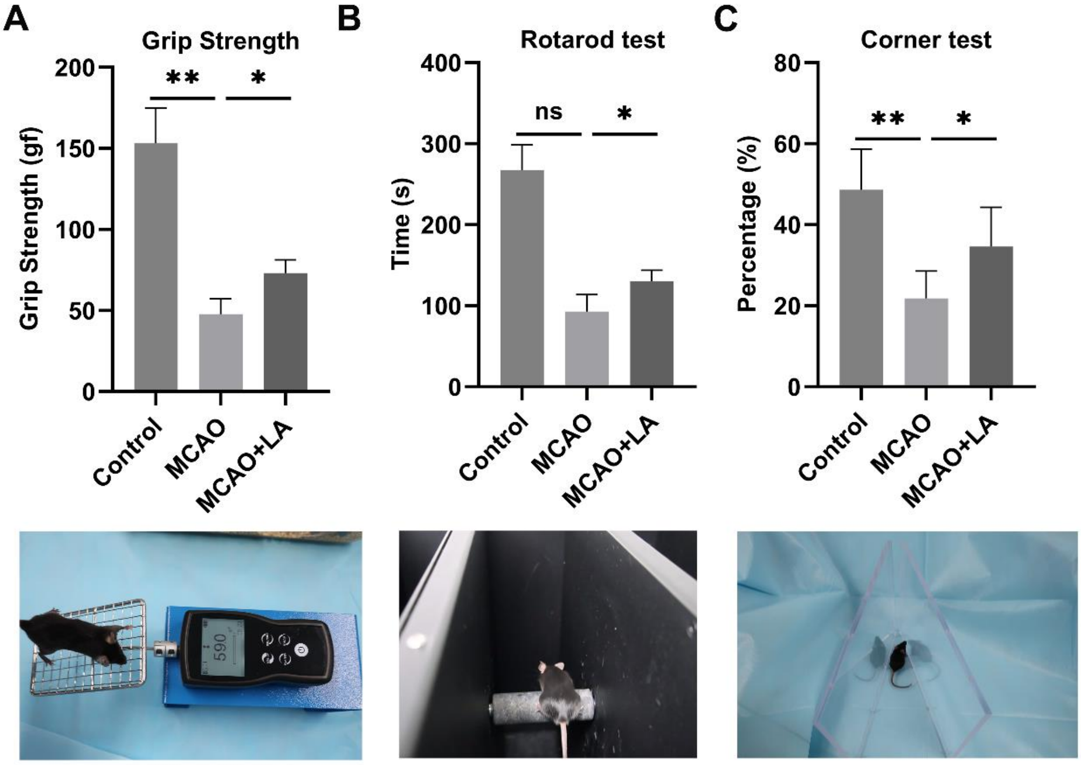

Supplementary Figure S1: Neurobehavioral evaluation of MCAO mice. (A) Grip Strength test. (B) Rotating rod experiment. (C) Corner experiment. (mean ± SD, $n = 3$, $^{*}P < 0.05$, $^{**}P < 0.01$).

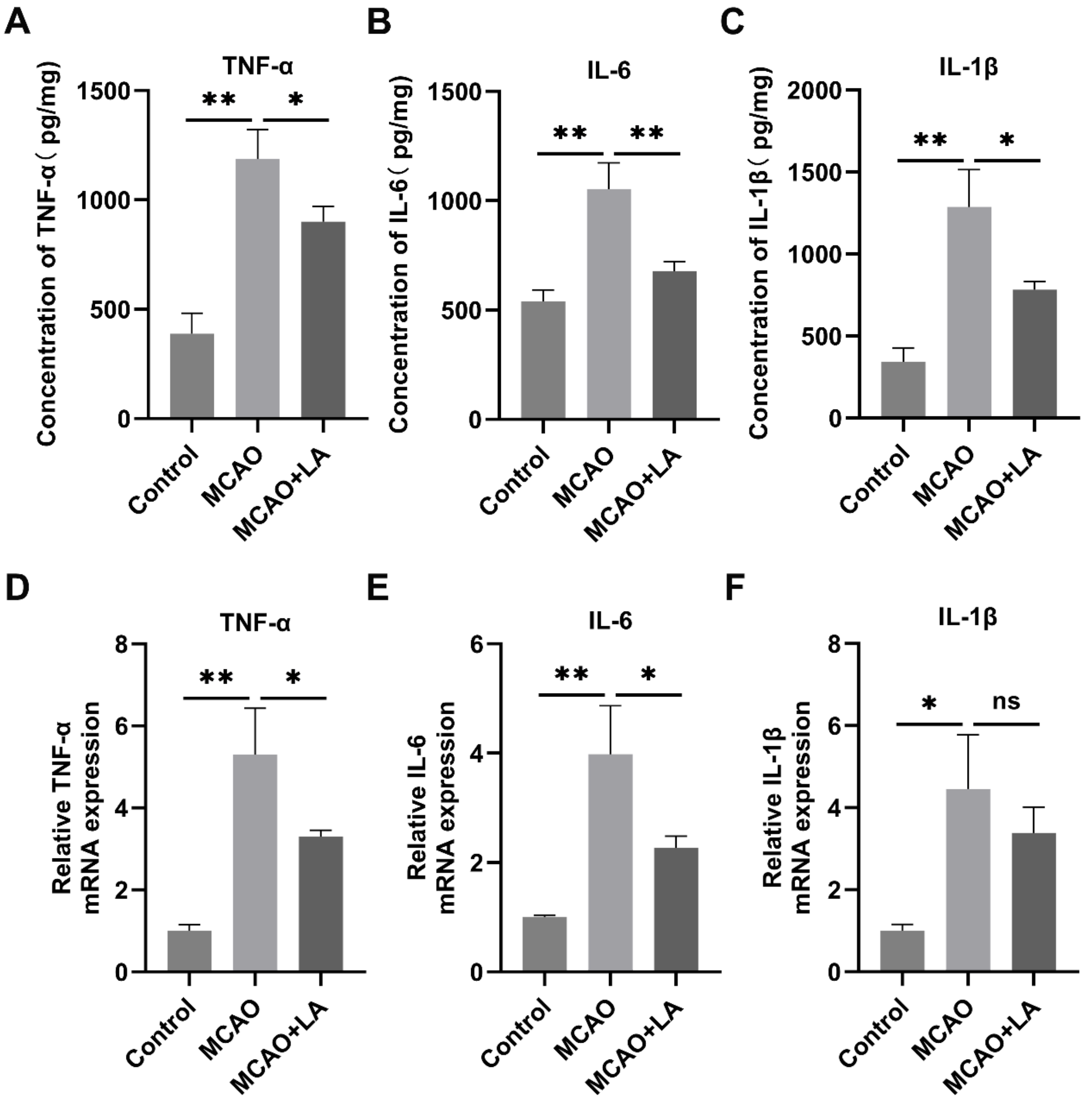

Supplementary Figure S2: The expression of inflammatory factors in the injured brain tissue of MCAO model mice. (A-C) Secreted cytokine levels of TNF-α, IL-6 and IL-1β were measured by ELISA in the injured brain tissue. (D-F) Secreted cytokine levels of TNF-α, IL-6 and IL-1β were measured by qPCR in the injured brain tissue. (mean ± SD, $n = 3$, $^{*}P < 0.05$, $^{**}P < 0.01$).

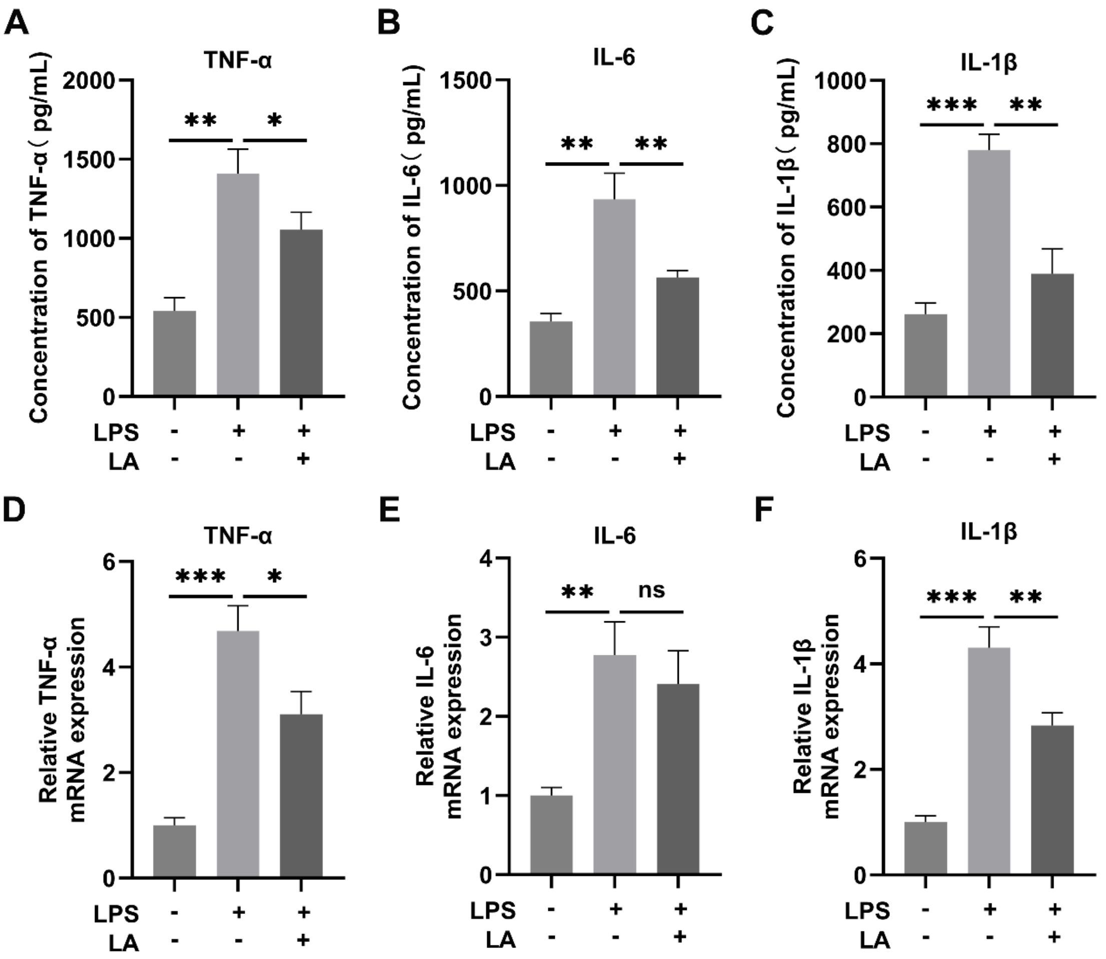

Supplementary Figure S3: The expression of inflammatory factors in primary microglia. (A-C) Secreted cytokine levels of TNF-α, IL-6 and IL-1β were measured by ELISA in primary microglia. (D-F) Secreted cytokine levels of TNF-α, IL-6 and IL-1β were measured by qPCR in primary microglia. (mean ± SD, n = 3, $^{*}P < 0.05$, $^{**}P < 0.01$, $^{***}P < 0.001$).

Supplementary Table S1: List of primers used in this study

TNF-α	*F*: CAGGCGGTGCCTATGTCTC
	R: CGATCACCCCGAAGTTCAGTAG
IL-6	*F*: CCAAGAGGTGAGTGCTTCCC
	R: CTGTTGTTCAGACTCTCTCCCT
IL-1β	*F*: GCAACTGTTCCTGAACTCAACT
	R: ATCTTTTGGGGTCCGTCAACT
β-actin	*F*: GGCTGTATTCCCCTCCATCG
	R: CCAGTTGGTAACAATGCCATGT
